# Supplementary material for: Acetylation dynamics and stoichiometry in Saccharomyces cerevisiae
Source: Mol Syst Biol. 2014 Jan 31;10(1):716. doi: 10.1002/msb.134766 (PMC4023402; doi:10.1002/msb.134766)
Supplement: Supplementary file 1 — Supplementary Figure 1 [file MSB-10-1-716-s009.pdf]

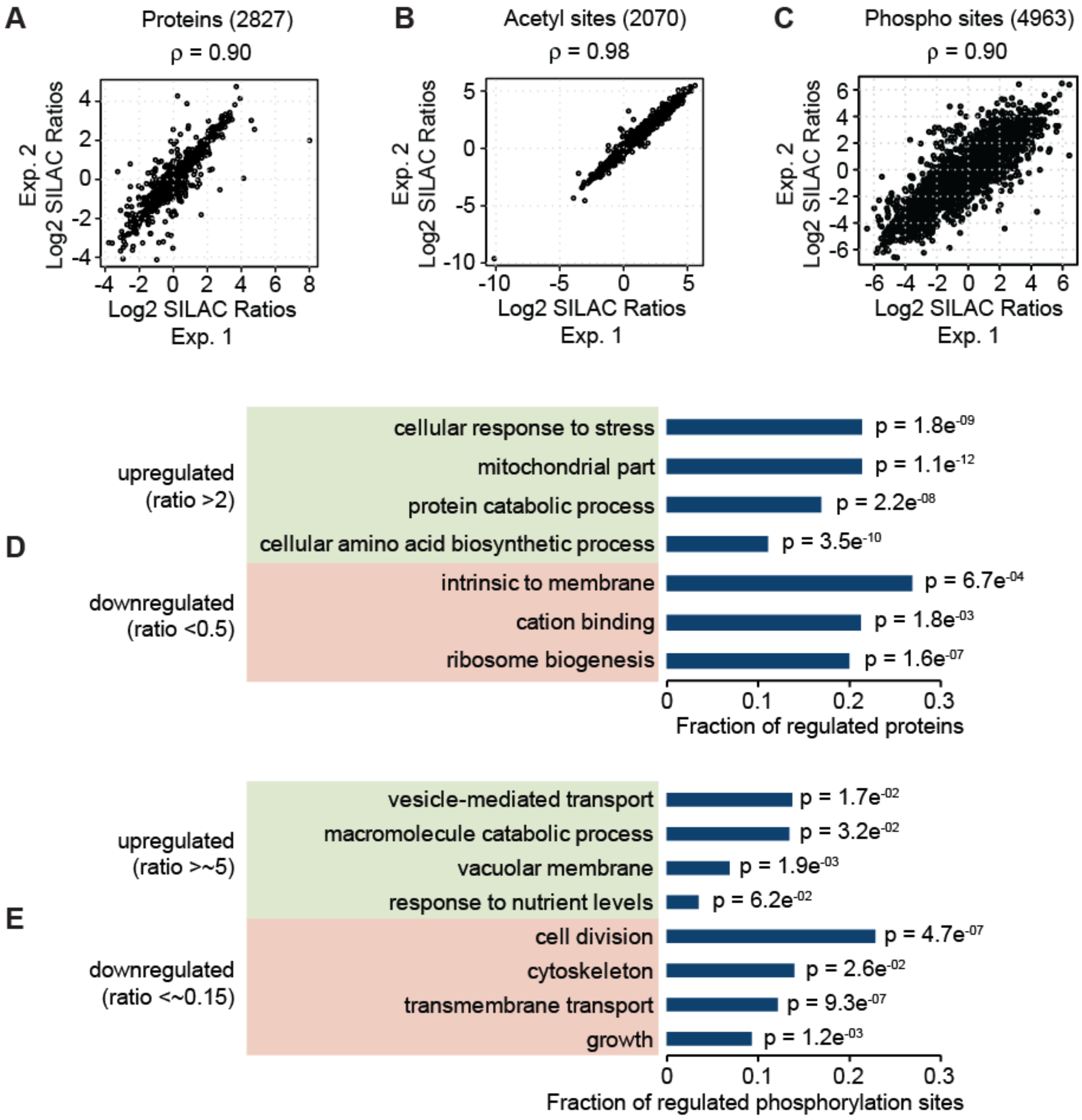

Figure S1. Proteomic analysis of stationary phase yeast. (**A-C**) Scatter plots showing the correlation between biological replicates (experiments 1 and 2, Exp. 1 and Exp. 2) comparing stationary phase to exponential phase yeast. The number of proteins or phosphorylation sites analyzed is indicated in parenthesis and the Spearman's rank correlation coefficient (ρ) is shown. (**D**) Gene ontology terms that are significantly more frequently associated with up- or downregulated proteins are shown. (**E**) Gene ontology terms that are significantly more frequently associated with up- or down-regulated phosphorylation sites are shown. Upregulated sites were determined by taking the sites with the highest ratios corresponding to 10% of the total sites (ratio >~5), downregulated sites were determined by taking sites with the lowest ratios corresponding to 10% of the total sites (ratio

<~0.15). For both C and D gene ontology enrichment and significance was determined using DAVID (Huang da et al, 2009) by comparing regulated proteins to all detected proteins.
