# Supplementary material for: Acetylation dynamics and stoichiometry in Saccharomyces cerevisiae
Source: Mol Syst Biol. 2014 Jan 31;10(1):716. doi: 10.1002/msb.134766 (PMC4023402; doi:10.1002/msb.134766)
Supplement: Supplementary file 2 — Supplementary Figure 2 [file MSB-10-1-716-s011.pdf]

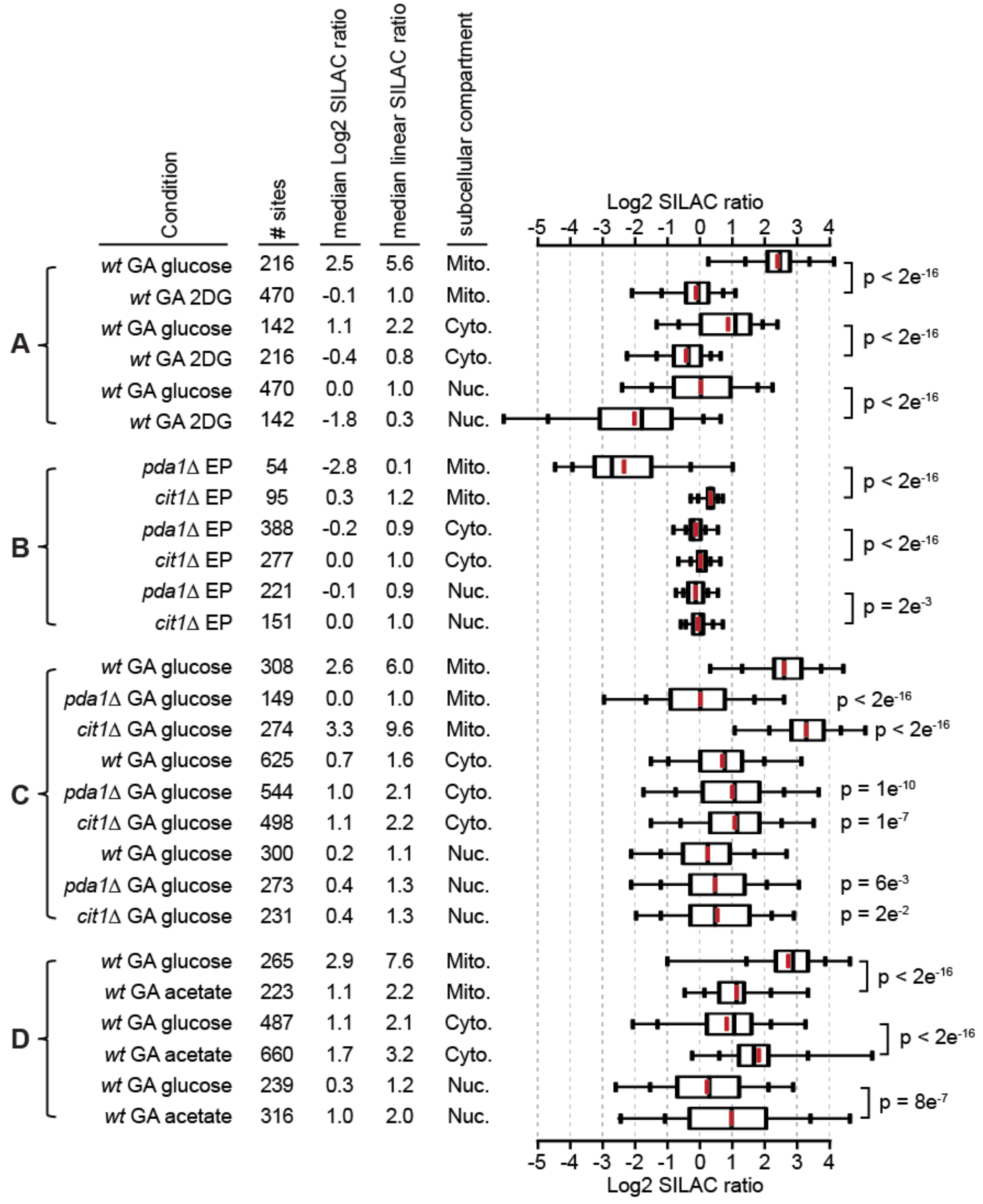

Figure S2. Quantitative analysis of acetylation dynamics in yeast. (**A-D**) The figure shows the conditions analyzed in each experiment, the cell type; wild-type (*wt*) or indicated mutant strains, the growth state; exponential phase (EP) or growth-arrested (GA), the number of acetylation sites analyzed (# sites), the median Log2 and linear SILAC ratios comparing the indicated condition to wild-type EP cells, and the subcellular localization of the analyzed acetylation sites on proteins localized to mitochondria (Mito.), the cytoplasm (Cyto.), or the nucleus (Nuc.). Cells were growth-arrested by transferring an exponential phase culture into media lacking lysine and containing the

indicated carbon sources, glucose, acetate, or 2-deoxy-D-glucose (2DG). The box plots show the SILAC ratios distributions comparing the indicated condition to wild-type EP cells, statistical significance was calculated by Wilcoxon test. (**B**) Increased acetylation requires glycolysis. Data is from two biological replicates. (**C**) Mitochondrial acetylation in exponentially growing cells requires Pda1. (**D**) Increased mitochondrial acetylation in growth-arrested cells is suppressed by loss of Pda1 and enhanced by loss of Cit1. Data from two biological replicates is shown, significant differences are relative to wild-type cells. (**E**) Acetate promotes cytoplasmic and nuclear acetylation. Data is from two biological replicates.
