# Supplementary material for: Acetylation dynamics and stoichiometry in Saccharomyces cerevisiae
Source: Mol Syst Biol. 2014 Jan 31;10(1):716. doi: 10.1002/msb.134766 (PMC4023402; doi:10.1002/msb.134766)
Supplement: Supplementary file 3 — Supplementary Figure 3 [file MSB-10-1-716-s013.pdf]

A

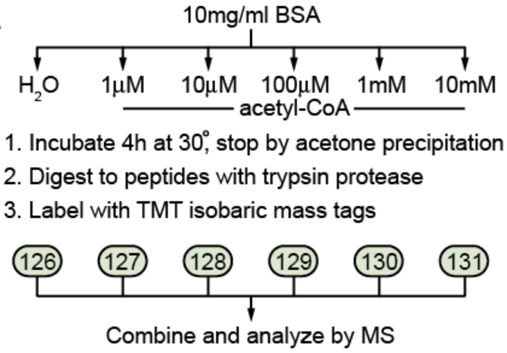

B

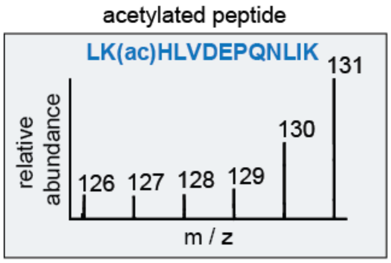

C

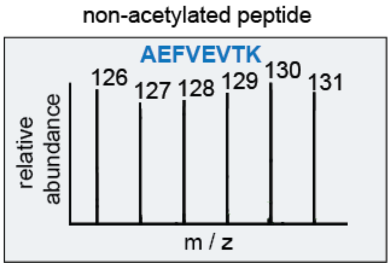

Figure S3. Nonenzymatic acetylation by acetyl-CoA. (**A**) Diagram showing the experimental conditions and quantitative method using TMT isobaric mass tags. (**B**) Plot showing a representative MS/MS scan of the indicated acetylated peptide. The region containing the TMT reporter ions is shown. (**C**) Plot showing a representative MS/MS scan and TMT reporter ions for an unmodified peptide as in panel B.
