# Supplementary material for: Acetylation dynamics and stoichiometry in Saccharomyces cerevisiae
Source: Mol Syst Biol. 2014 Jan 31;10(1):716. doi: 10.1002/msb.134766 (PMC4023402; doi:10.1002/msb.134766)
Supplement: Supplementary file 4 — Supplementary Figure 4 [file MSB-10-1-716-s015.pdf]

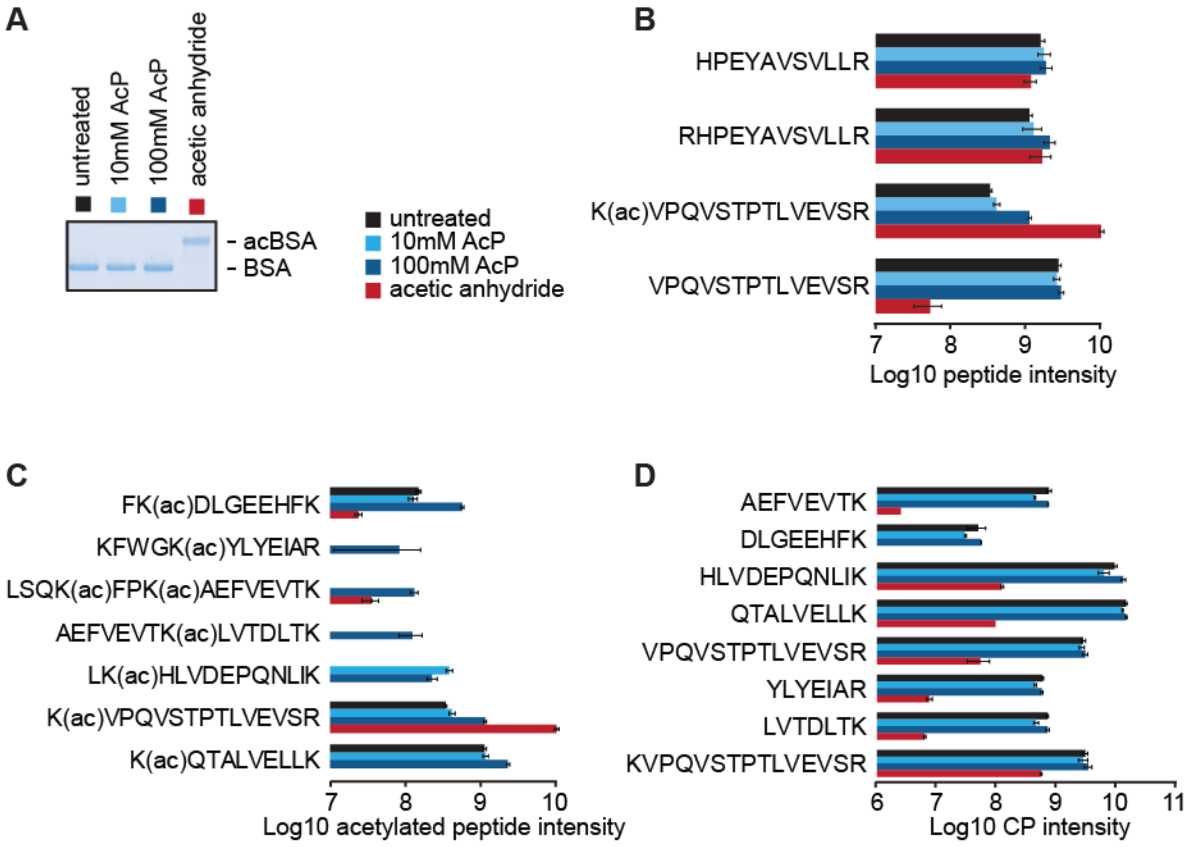

Figure S4. Validating partial chemical acetylation by acetyl-phosphate. (**A**) Partial chemical acetylation by acetyl-phosphate (AcP) and comprehensive acetylation by acetic anhydride. Chemically acetylated (ac) or untreated BSA was separated by denaturing polyacrylamide gel electrophoresis and stained with Coomassie blue. (**B**) Substantially increased acetylation causes a proportional reduction in the abundance of unmodified corresponding peptides (CPs). The column graph shows peptide signal intensities (error bars = +/- standard deviation of triplicate measurements) of the indicated peptides isolated from untreated (black), 10mM AcP (light blue), 100mM AcP (dark blue), and acetic anhydride (red)-treated BSA as shown in panel A. Acetylated lysine is indicated by an (ac) following the modified lysine residue. (**C**) Acetylated peptides identified in BSA. The bar graph shows acetylated peptide signal intensities (error bars = +/- standard deviation of triplicate measurements) for the indicated acetylated peptides. Since acetic anhydride comprehensively acetylates lysine residues, the trypic digestion at all lysine residues is inhibited; therefore acetylation is reduced or not identified. However, digestion of the peptide K(ac)VPQVSTPTLVEVSR is unaffected as this peptide is flanked by arginine residues. (**D**) CP abundance is reduced after near-comprehensive acetylation by acetic anhydride. The bar graph shows non-acetylated peptide signal intensities (error bars = +/- standard deviation) for the

indicated CPs covering the acetylation sites shown in panel B. Note that 100mM AcP does not result in reduced abundance of CPs.
