# Supplementary material for: Acetylation dynamics and stoichiometry in Saccharomyces cerevisiae
Source: Mol Syst Biol. 2014 Jan 31;10(1):716. doi: 10.1002/msb.134766 (PMC4023402; doi:10.1002/msb.134766)
Supplement: Supplementary file 5 — Supplementary Figure 5 [file MSB-10-1-716-s016.pdf]

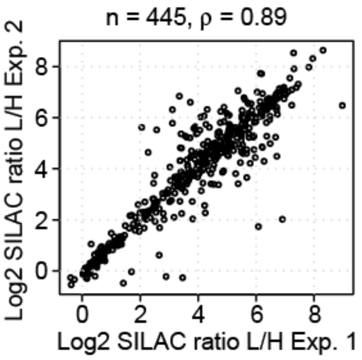

Figure S5. Yeast acetylation sites are sensitive to treatment with acetyl-phosphate (AcP). 100mM AcP causes similar acetylation changes in two independent experimental replicates (Exp. 1 and Exp. 2). The scatterplot shows the SILAC L/H ratios for acetylation sites after treatment with AcP. The number (n) of sites analyzed and Spearman's correlation coefficient (ρ) is shown.
