# Supplementary material for: Acetylation dynamics and stoichiometry in Saccharomyces cerevisiae
Source: Mol Syst Biol. 2014 Jan 31;10(1):716. doi: 10.1002/msb.134766 (PMC4023402; doi:10.1002/msb.134766)
Supplement: Supplementary file 6 — Supplementary Figure 6 [file MSB-10-1-716-s017.pdf]

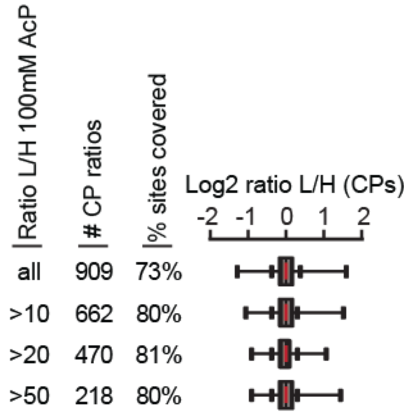

Figure S6. Unmodified corresponding peptide (CP) abundance is unaffected by treatment with AcP. The box plots show the distributions of CPs covering the indicated categories of acetylation sites shown in Figure 5B. Percent sites covered indicates the fraction of sites in each category for which at least one CP was quantified.
