# Supplementary material for: Acetylation dynamics and stoichiometry in Saccharomyces cerevisiae
Source: Mol Syst Biol. 2014 Jan 31;10(1):716. doi: 10.1002/msb.134766 (PMC4023402; doi:10.1002/msb.134766)
Supplement: Supplementary file 7 — Supplementary Figure 7 [file MSB-10-1-716-s018.pdf]

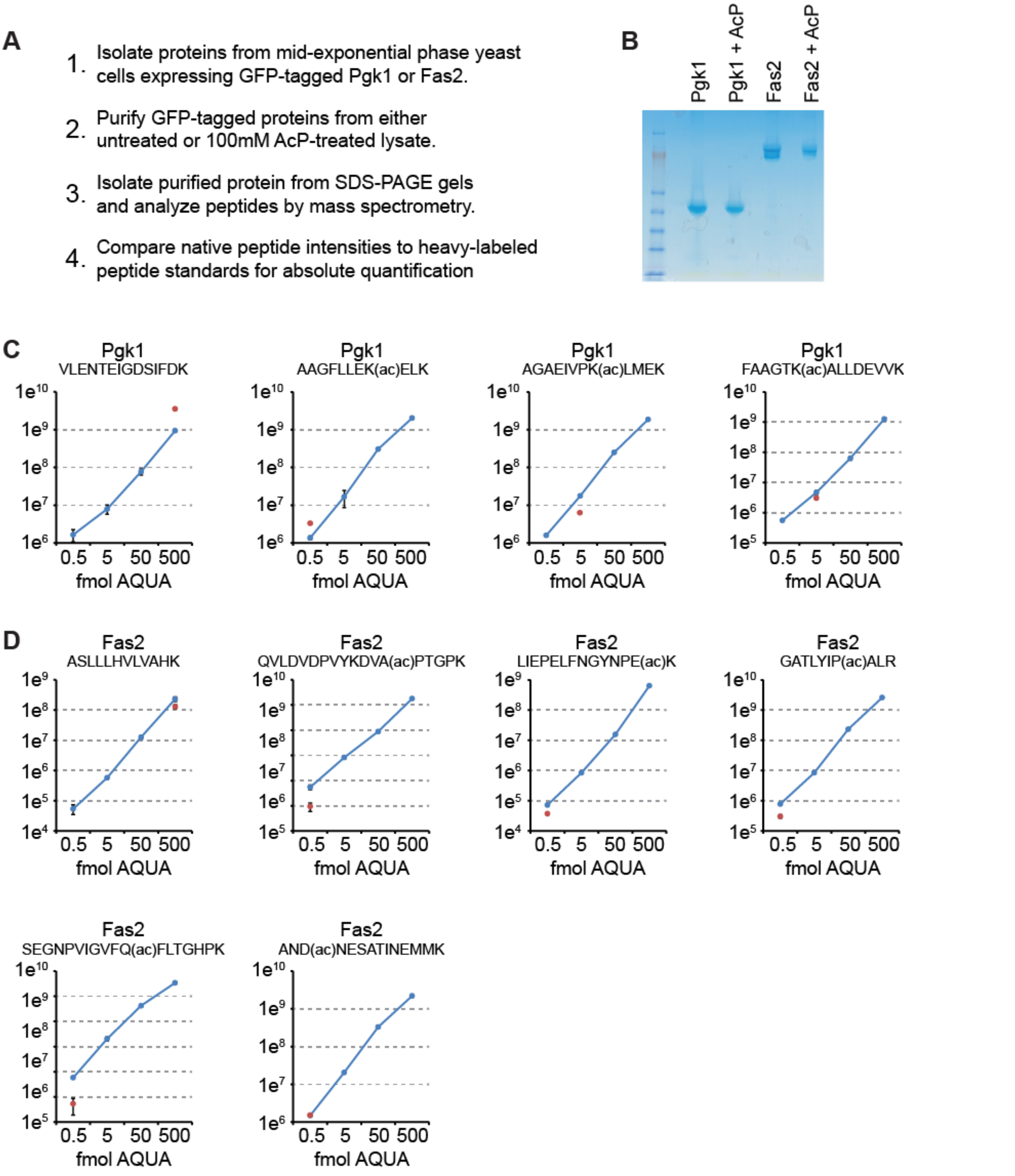

Figure S7. Determining absolute acetylation stoichiometry using AQUA. (**A**) Outline of the experimental approach for absolute quantification (AQUA) of acetylated peptides from Pgk1 and Fas2. (**B**) SDS-PAGE gel showing the purified proteins used in the AQUA analysis. (**C-D**) Line plots showing AQUA peptide intensities at the indicated concentrations (Blue) and the intensity of the native peptide (Red spot). Native peptides were mixed with AQUA standards immediately before

analysis by mass spectrometry (MS). The native peptide concentration was determined by comparing the peptide intensity to the AQUA peptide concentration with the nearest intensity, as shown. Data is from three technical replicates (error bars indicate standard deviation of these replicates). Two biological replicates were performed with comparable results. The protein name and peptide sequence analyzed is shown above each plot.
