# Supplementary material for: Acetylation dynamics and stoichiometry in Saccharomyces cerevisiae
Source: Mol Syst Biol. 2014 Jan 31;10(1):716. doi: 10.1002/msb.134766 (PMC4023402; doi:10.1002/msb.134766)
Supplement: Supplementary file 8 — Supplementary Figure 8 [file MSB-10-1-716-s019.pdf]

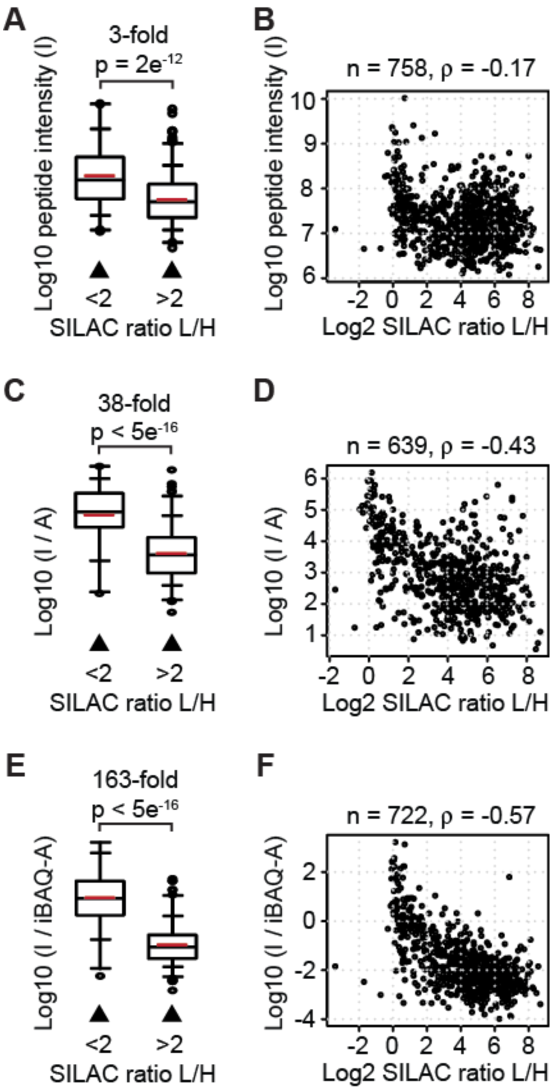

Figure S8. Acetylation stoichiometry is proportional to abundance-corrected acetylated peptide intensity. (**A**) Acetylated peptides with high stoichiometry, AcP-insensitive sites (SILAC ratio L/H <2) have a significantly higher median intensity than peptides with low-stoichiometry, AcP-sensitive sites (SILAC ratio L/H >2). The box plots show the distributions of peptide intensities and the fold difference in median intensity is shown, significance was determined by Wilcoxon test. (**B**) Scatterplot showing the correlation between peptide intensity (I) and AcP-sensitivity (Log2 SILAC ratio L/H). Number (n) of sites analyzed and Spearman's correlation coefficient (ρ) are shown. (**C**) Acetylated peptides with AcP-insensitive sites have a significantly higher median abundance-corrected intensity (I/A) than peptides with AcP-sensitive sites. The box plots show the distributions of I/A values (based on previously determined protein abundances expressed as copies per cell (Ghaemmaghami et al, 2003)) and the fold difference in I/A is shown. Significance was determined

by Wilcoxon test. (**D**) Scatterplot showing the correlation between I/A and AcP-sensitivity (Log2 SILAC ratio L/H). (**E**) iBAQ-based abundance-corrected intensity (I/iBAQ-A) provides the best distinction between AcP-insensitive and AcP-sensitive sites. The box plots show the distributions of I/iBAQ-A values and the fold difference in I/iBAQ-A is shown. Significance was determined by Wilcoxon test. (**F**) Scatterplot showing the correlation between I/iBAQ-A and AcP-sensitivity (Log2 SILAC ratio L/H).
