# Supplementary material for: Acetylation dynamics and stoichiometry in Saccharomyces cerevisiae
Source: Mol Syst Biol. 2014 Jan 31;10(1):716. doi: 10.1002/msb.134766 (PMC4023402; doi:10.1002/msb.134766)
Supplement: Supplementary file 9 — Supplementary Figure 9 [file MSB-10-1-716-s020.pdf]

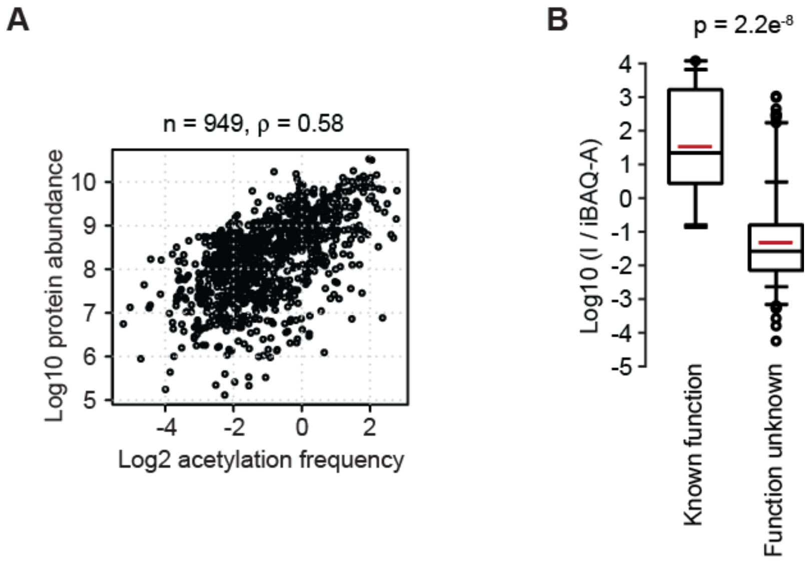

Figure S9. Functional characterization of acetylation stoichiometry. (**A**) The frequency of detected acetylation sites is proportional to protein abundance. The scatter plot shows the iBAQ protein abundance and acetylation frequency (sites/100 amino acids) for the indicated number (n) of acetylated proteins. The Spearman's correlation coefficient (ρ) is shown. (**B**) Known functional sites have significantly higher iBAQ-based abundance corrected peptide intensities (I/iBAQ-A). The box plots show the distributions of I/iBAQ-A for sites with known functions and for sites where the functional consequence of acetylation is unknown. Significance (p) was calculated by Wilcoxen test.
