# Supplementary material for: Acetylation dynamics and stoichiometry in Saccharomyces cerevisiae
Source: Mol Syst Biol. 2014 Jan 31;10(1):716. doi: 10.1002/msb.134766 (PMC4023402; doi:10.1002/msb.134766)
Supplement: Supplementary file 10 — Supplementary Table 1 [file MSB-10-1-716-s021.pdf]

Table	SILAC-L	SILAC-M	SILAC-H	Total sites	Decoy sites	FDR
**S2**	BY4742 Lys^0 EP		BY4742 Lys^8 SP	2,784	10	0.36%
**S3**	BY4742 Lys^0 EP		BY4742 Lys^8 SP	3,327 Proteins	1	0.03%
**S4**	BY4742 Lys^0 EP		BY4742 Lys^8 SP	6,260 Phospho.	7	0.11%
**S5**	BY4742 Lys^0 EP	BY4742 Lys^4 GA + glucose	BY4742 Lys^8 GA + 2DG	1,484	5	0.34%
**S6**	BY4742 Lys^0 EP		*pda1*Δ Lys^8 EP	1,254	3	0.24%
**S6**	BY4742 Lys^0 EP		*cit1*Δ Lys^8 EP	1,254	3	0.24%
**S7**	BY4742 Lys^0 EP	BY4742 Lys^4 GA	*pda1*Δ Lys^8 GA	1,988	8	0.40%
**S7**	BY4742 Lys^0 EP	BY4742 Lys^4 GA	*cit1*Δ Lys^8 GA	1,988	8	0.40%
**S8**	BY4742 Lys^0 EP		BY4742 Lys^8 GA + 2% Glu. or 2% Ace.	2,139	8	0.37%
**S9**	BY4742 Lys^0 EP		BY4742 Lys^8 EP	9,666	29	0.30%

Table S1. Quantitative MS experiments performed in this study. The yeast strain used and growth phase (exponential phase (EP), stationary phase (SP), or growth-arrested (GA)), SILAC labeling scheme (L=light, M=medium, H=heavy), total sites identified, and the number of sites identified from a reversed sequence, decoy database is shown. The estimated false discovery rate (FDR) is calculated from the frequency of decoy sites identified in the data set (Elias & Gygi, 2007).
