# Supplementary material for: Acetylation dynamics and stoichiometry in Saccharomyces cerevisiae
Source: Mol Syst Biol. 2014 Jan 31;10(1):716. doi: 10.1002/msb.134766 (PMC4023402; doi:10.1002/msb.134766)
Supplement: Supplementary file 19 — Supplementary Data [file MSB-10-1-716-s031.pdf]

Validating partial chemical acetylation by acetyl-phosphate (supplemental text)

Bovine serum albumin (BSA) was chemically acetylated by treating with two concentrations of acetyl-phosphate (AcP) or with acetic anhydride. Treatment with AcP did not alter the electrophoretic mobility of BSA, while acetic anhydride resulted in uniformly retarded mobility in a denaturing polyacrylamide gel, suggesting comprehensive chemical acetylation (**Figure S4A**). Control and *in vitro* acetylated BSA was digested to peptides with trypsin protease and analyzed in triplicate by MS. Trypsin cleaves polypeptide chains at the C-terminus of lysine and arginine residues. Acetylation of lysine blocks proteolytic digestion by trypsin, however, arginine-flanked peptides should be unaffected by acetylation. The unmodified, arginine-flanked peptides HPEYAVSVLLR and RHPEYAVSVLLR had similar peptide intensities regardless of whether BSA was chemically acetylated, indicating that a similar amount of BSA was analyzed in each sample (**Figure S4B**). The arginine-flanked, acetylated (ac) peptide K(ac)VPQVSTPTLVEVSR was ~3.4-fold more abundant after treatment with 100mM AcP and ~30-fold more abundant after treatment with acetic anhydride. The abundance of the CP for the acetylated peptide, VPQVSTPTLVEVSR, was substantially reduced (~50-fold) in acetic anhydride-treated BSA, but was unaffected by treatment with AcP (**Figure S4B**). Based on the above abundance changes, we estimated the stoichiometry of acetylation at this position to be ~3% in untreated BSA and ~98% in acetic anhydride treated BSA (see below). Even though treatment with 100mM AcP caused acetylation at 7 sites (**Figure S4C**), we were unable to detect a significant reduction in the abundance of eight different CPs covering these sites, while acetic anhydride resulted in a median 100-fold reduction in the abundance of these CPs (**Figure S4D**). These data indicate that treatment with AcP resulted in partial chemical acetylation of lysine residues, which did not cause a detectable reduction in CP abundance. In contrast, near-comprehensive chemical acetylation by acetic anhydride resulted in markedly reduced abundance of CPs.

Estimating acetylation stoichiometry based on unmodified corresponding peptides (supplemental text)

We estimated acetylation stoichiometry on BSA using the method described in (Olsen et al, 2010). In order to perform this calculation we used the ratio changes between untreated and acetic anhydride treated BSA for acetylated peptide abundance (x, 30.26), unmodified corresponding peptide abundance (y, 0.019), and protein abundance (z, 1). The proportion acetylated in untreated BSA was ((z-y)/(x-z) = a) and the proportion acetylated of acetic anhydride-treated BSA was (x*(z-

y)/y*(x-z) = b). Acetylation stoichiometry in untreated BSA was (a/(1+a) = 3.4%) and acetylation stoichiometry in acetic anhydride-treated BSA was (b/(b+1) = 98.2%).
