# Supplementary material for: Acetylation dynamics and stoichiometry in Saccharomyces cerevisiae
Source: Mol Syst Biol. 2014 Jan 31;10(1):716. doi: 10.1002/msb.134766 (PMC4023402; doi:10.1002/msb.134766)
Supplement: Supplementary file 20 — Supplementary Legends [file MSB-10-1-716-s032.pdf]

Supplementary Information

Acetylation dynamics and stoichiometry in *Saccharomyces cerevisiae*

Brian T. Weinert[1], Vytautas Iesmantavicius[1], Tarek Moustafa[2], Christian Schölz[1], Sebastian A. Wagner[1], Christoph Magnes[3], Rudolf Zechner[2], and Chunaram Choudhary[1,*]

[1]The NNF Center for Protein Research, Faculty of Health Sciences, University of Copenhagen, Blegdamsvej 3B, DK-2200 Copenhagen, Denmark

[2]Institute of Molecular Biosciences, University of Graz, Heinrichstrasse 31, A-8010 Graz, Austria

[3]HEALTH – Institute for Biomedicine and Health Sciences, Joanneum Research, Elisabethstrasse 5, A-8010 Graz, Austria

*Correspondence to: chuna.choudhary@cpr.ku.dk, Phone: +45 35 32 50 20

Table of contents

Figure S1. Proteomic analysis of stationary phase yeast.

Figure S2. Quantitative analysis of acetylation dynamics in yeast.

Figure S3. Nonenzymatic acetylation by acetyl-CoA.

Figure S4. Validating partial chemical acetylation by acetyl-phosphate.

Supplemental text. Validating partial chemical acetylation by acetyl-phosphate.

Supplemental text. Estimating acetylation stoichiometry based on unmodified corresponding peptides.

Figure S5. Yeast acetylation sites are sensitive to treatment with acetyl-phosphate.

Figure S6. Unmodified corresponding peptide (CP) abundance is unaffected by treatment with AcP.

Figure S7. Determining absolute acetylation stoichiometry using AQUA.

Figure S8. Acetylation stoichiometry is proportional to abundance-corrected acetylated peptide intensity.

Figure S9. Functional characterization of acetylation stoichiometry.

Table S1. Quantitative MS experiments performed in this study.

Supplemental References

Elias JE, Gygi SP (2007) Target-decoy search strategy for increased confidence in large-scale protein identifications by mass spectrometry. *Nature methods* **4:** 207-214

Ghaemmaghami S, Huh WK, Bower K, Howson RW, Belle A, Dephoure N, O'Shea EK, Weissman JS (2003) Global analysis of protein expression in yeast. *Nature* **425:** 737-741

Huang da W, Sherman BT, Lempicki RA (2009) Systematic and integrative analysis of large gene lists using DAVID bioinformatics resources. *Nat Protoc* **4:** 44-57

Olsen JV, Vermeulen M, Santamaria A, Kumar C, Miller ML, Jensen LJ, Gnad F, Cox J, Jensen TS, Nigg EA, Brunak S, Mann M (2010) Quantitative phosphoproteomics reveals widespread full phosphorylation site occupancy during mitosis. *Science signaling* **3:** ra3
